# Supplementary material for: Costs and cost-effectiveness of an infection prevention bundle to reduce neonatal sepsis and mortality in Zambia: The Sepsis Prevention in Neonates in Zambia (SPINZ) trial
Source: PLOS Glob Public Health. 2026 Jul 15;6(7):e0006016. doi: 10.1371/journal.pgph.0006016 (PMC13372121; doi:10.1371/journal.pgph.0006016)
Supplement: S1 Text — (DOC) [file pgph.0006016.s001.doc]

**Supplemental Table 1. Pre-intervention demographics and outcomes for SPINZ study by month.**

| **Study month** | **Total enrolled** | **Gender (Male, %)** | **Inborn**  **(n, %)** | **Birth weight**  **(median kg, IQR)** | **Length of stay (median days, IQR)** | **Cases of suspected sepsis**  **(n, %)** | **Lab-confirmed BSI (of those with suspected sepsis)**  **(n, %)** | **Deaths**  **(n, %)** |
| --- | --- | --- | --- | --- | --- | --- | --- | --- |
| Sep 2015 | 88 | 42 (48) | 70 (80) | 2.70 (1.50-3.20) | 6.5 (4-9) | 67 (76) | 28 (42) | 38 (43) |
| Oct 2015 | 90 | 47 (52) | 66 (73) | 2.81 (2.20-3.20) | 6 (4-10) | 86 (96) | 27 (31) | 26 (29) |
| Nov 2015 | 183 | 98 (54) | 144 (79) | 2.50 (1.60-3.06) | 6 (4-10) | 140 (77) | 65 (46) | 60 (33) |
| Dec 2015 | 90 | 44 (49) | 79 (88) | 2.50 (1.60-3.10) | 6 (4-10) | 59 (66) | 29 (49) | 30 (33) |
| Jan 2016 | 149 | 82 (55) | 103 (69) | 2.40 (1.60-3.20) | 6 (4-10) | 80 (54) | 31 (39) | 48 (32) |
| Feb 2016 | 182 | 101 (55) | 136 (75) | 2.30 (1.50-3.03) | 6 (4-10) | 115 (63) | 32 (28) | 38 (21) |
| Mar 2016 | 191 | 106 (56) | 138 (73) | 2.50 (1.60-3.20) | 6 (3-10) | 94 (49) | 29 (31) | 43 (23) |
| **Total** | **973** | **520 (53)** | **736 (76)** | **2.52 (1.60-3.10)** | **6 (4-10)** | **641 (66)** | **241 (38)** | **283 (29)** |
