# Supplementary material for: Costs and cost-effectiveness of an infection prevention bundle to reduce neonatal sepsis and mortality in Zambia: The Sepsis Prevention in Neonates in Zambia (SPINZ) trial
Source: PLOS Glob Public Health. 2026 Jul 15;6(7):e0006016. doi: 10.1371/journal.pgph.0006016 (PMC13372121; doi:10.1371/journal.pgph.0006016)
Supplement: S2 Text — (DOC) [file pgph.0006016.s002.doc]

**Supplemental Table 2. Demographics and outcomes for SPINZ study by intervention month**

| **Study month** | **Total enrolled** | **Gender (Male, %)** | **Inborn**  **(n, %)** | **Birth weight (median kg, IQR)** | **Length of stay (median days, IQR)** | **Cases of suspected sepsis**  **(n, %)** | **Lab-confirmed BSI (of those with suspected sepsis)**  **(n, %)** | **Deaths**  **(n, %)** |
| --- | --- | --- | --- | --- | --- | --- | --- | --- |
| Jun 2016 | 183 | 93 (51) | 155 (85) | 2.40 (1.60-3.10) | 6 (3-11) | 103 (56) | 17 (17) | 35 (19) |
| Jul 2016 | 194 | 117 (60) | 146 (75) | 2.25 (1.50-3) | 4 (2-8) | 94 (48) | 17 (18) | 67 (35) |
| Aug 2016 | 237 | 125 (52) | 163 (69) | 2.10 (1.45-3) | 5 (3-10) | 85 (36) | 14 (16) | 53 (22) |
| Sep 2016 | 243 | 131 (54) | 166 (68) | 2.60 (1.60-3.20) | 4 (2-8) | 72 (30) | 26 (36) | 70 (29) |
| Oct 2016 | 260 | 141 (54) | 158 (61) | 2.54 (1.70-3.10) | 5 (2-8) | 55 (21) | 4 (7) | 63 (24) |
| Nov 2016 | 251 | 151 (61) | 158 (63) | 2.40 (1.60-3) | 4 (2-8) | 68 (27) | 6 (9) | 60 (24) |
| Dec 2016 | 96 | 47 (49) | 67 (70) | 2.06 (1.40-3.16) | 3 (2-7) | 23 (24) | 5 (22) | 26 (27) |
| Jan 2017 | 200 | 112 (56) | 130 (65) | 2.20 (1.50-3) | 5 (3-10) | 77 (39) | 29 (38) | 53 (27) |
| Feb 2017 | 203 | 105 (52) | 139 (68) | 2.20 (1.50-3) | 6 (4-10) | 84 (41) | 21 (25) | 56 (28) |
| Mar 2017 | 168 | 84 (50) | 111 (66) | 2.46 (1.61-3.15) | 6 (3-11) | 30 (18) | 14 (47) | 31 (18) |
| **Total** | **2035** | **1106 (54)** | **1393 (68)** | **2.40 (1.54-3.04)** | **5 (3-9)** | **691 (34)** | **153 (22)** | **514 (25)** |
